# Six months of different exercise type in sedentary primary schoolchildren: impact on physical fitness and saliva microbiota composition

**DOI:** 10.3389/fnut.2024.1465707

**Published:** 2024-10-24

**Authors:** Annamaria Mancini, Daniela Vitucci, Vito Alessandro Lasorsa, Corrado Lupo, Paolo Riccardo Brustio, Mario Capasso, Stefania Orrù, Alberto Rainoldi, Federico Schena, Pasqualina Buono

**Affiliations:** ^1^Department of Medicine, Movement Sciences and Wellness, University Parthenope, Naples, Italy; ^2^CEINGE-Biotecnologie Avanzate “Franco Salvatore”, Napoli, Italy; ^3^Department of Medical Sciences, University of Turin, Turin, Italy; ^4^Department of Clinical and Biological Sciences, University of Turin, Turin, Italy; ^5^Department of Molecular Medicine and Medical Biotechnologies, University of Naples Federico II, Naples, Italy; ^6^Department of Neuroscience, Biomedicine and Movement, University of Verona, Verona, Italy

**Keywords:** saliva microbiota, schoolchildren, physical fitness, exercise, health

## Abstract

**Introduction:**

Lifestyle influences microbiota composition. We previously reported a healthier microbiota composition in saliva from active schoolchildren compared to sedentary. In the present study, we evaluated the effects of 6 months of different exercise types on physical fitness and saliva microbiota composition in 8-11-years-old sedentary schoolchildren.

**Methods:**

Sixty-four sedentary children from five primary schools in Turin, Italy, were divided into three groups: one continued normal curricular activity while two underwent different exercise protocols for 6 months. The Structured Exercise (Sa) group did 2 h per week of muscle activation, strength and coordination exercises supervised by a kinesiologist. The Daily Mile (Dm) group did 1 h per week of Sa plus 15 min of walking/running outdoors four times a week, supervised by a class teacher; control group (Ct) did 2 h a week of curricular exercise supervised by a class teacher. Physical fitness was evaluated before and after the intervention. Saliva samples were collected post-intervention in all participants and analyzed using PCR amplification of 16S rRNA bacterial genes. The Amplicon Sequence Variants were filtered, decontaminated, and phylogenetically classified using DADA2 software. Differential abundance analysis of microbiome taxa and pathway data was conducted using the LEfSe algorithm and PICRUSt.

**Results:**

The Sa group showed better performances in lower limb power and sprint performance while both the Sa and Dm groups improved in endurance and balance at the end of the intervention; only balance resulted slightly improved in the Ct group. Among the genera differently enriched in saliva after the training intervention, we found that the *Prevotella*, the *Dubosiella* and the *Family XIII AD3011 group* were the most abundant in the Sa group; differently, the *Neisseria* and the *Abiotrophia* in Ct group. Four species showed significant the *Prevotella melaninogenica* and the *Prevotella nanceiensis* were more abundant in the Sa, conversely, *Gemella sanguinis* was enriched in Dm and *Abiotrophia defectiva* in Ct saliva group.

**Conclusion:**

We demonstrated that Sa and Dm, not curricular exercise, improve the physical fitness components in sedentary schoolchildren correlated to health and promote an enrichment in saliva microbiota species associated to a healthier profile.

## 1 Introduction

The human microbiota is an ecosystem of trillions of microorganisms comprising bacteria, viruses, fungi and archaea that inhabit our body (1, 2). The microbiota genome, called microbiome, is considered ‘our second genome’ and ‘our last organ’ due to its important role in human health and wellness (2–4). The human microbiota was mainly located in the gut, although it was abundant in other organs and tissues including oral cavity, lung, vagina and skin; the composition differs from site to site. The gut microbiota composition is the most studied, to date, in association to human health (5, 6). Gut bacteria have several functions, such as food fermentation, protection from pathogens, immune response stimulation, uptake of nutrients that would otherwise be unavailable to the host, production of vitamins, such as vitamin K and the B vitamins. Scientific evidence indicate that different environmental factors, as pollutants, antibiotics, stress, and an unhealthy lifestyle, as unbalanced diet, sedentary lifestyle, negatively impact on the composition and function of the gut microbiota leading to disruption of microbial homeostasis (i.e., dysbiosis) (7–10). Microbial dysbiosis have been associated to the development of different pathologies, including obesity (10–13), type 2 diabetes (11, 14) and some type of cancer (11, 15). The oral microbiota represents the second largest microbial community in humans (6, 16). It has been demonstrated that oral microbiota composition affects not only the oral but also the systemic health contributing to colonization resistance (17), nutrient digestion (18) and immune response (19, 20). Scientific evidence have been provided on the association between the oral microbiome composition and different diseases including cardiovascular disease (21, 22), atherosclerosis, obesity (22–25), colorectal cancer (20, 26–28), pancreatic cancer (26, 29), squamous cell cancer of the head and neck (20, 27). Therefore, there is a growing interest in determining the composition of the “healthy” oral microbiome and the factors that could influence its composition (2, 30).

Physical activity (PA) is one of the main environmental factors modulating the composition of the intestinal microflora (11, 12, 14, 31, 32), leading to an increase in the diversity of the microbiota (32, 33). PA, by stimulating bacterial proliferation, has been shown to improve intestinal barrier function and modulation of mucosal immunity leading to a reduction of the incidence of metabolic diseases and obesity (11, 34). Furthermore, it has been demonstrated that there is a positive association between PA levels and biodiversity of the microbiota. Clauss and colleagues reported that moderate resistance exercise reduces inflammation, improves body composition and positively influences the enrichment and biodiversity of the gut microbiome in association to improved human metabolic health when intensity is controlled (35). In a recent meta-analysis by Pérez-Prieto and colleagues, a positive association was reported between PA levels and the relative abundance of short-chain fatty acid-(SCFA) producing bacteria; furthermore, in the same meta-analysis it was evidenced that athletes appear to have a richer gut microbiome than non-athletes (2, 36). Understanding the dynamic interaction between exercise and microbiota is important not only to elucidate the complex mechanisms that regulate human health, but also to develop targeted interventions to promote health and well-being in different age groups. The knowledge of how physical activity can influence the microbiota composition in childhood and adolescent becomes particularly crucial as it could offer the possibility of studying the potential implications of exercise/sport practice on adult health. Recent studies have shown that gut microbiota composition can be modulated by lifestyle and PA in obese children (37–40). We previously reported that active primary schoolchildren had an enrichment in saliva composition of bacterial species, such as *Prevotella nigrescens*, *Collinsella aerofaciens*, *Simonsiella muelleri* and *Parabacteroides merdae*, associated with a healthier profile as compared to sedentary children that presented an enrichment of species, such as *Gemella parahaemolysans*, *Prevotella aurantiaca*, *Prevotella pallens* and *Neisseria mucosa*, associated to human diseases (41). The aim of this paper was to evaluate the effects of 6 months (6-mo) of different exercise type, Structured (Sa) or Daily Mile (Dm) compared to curricular (Ct) on physical fitness components associated to health and on saliva microbiota composition in the 8-11-years-old schoolchildren previously classified as sedentary.

## 2 Materials and methods

### 2.1 Participants

Sixty-four schoolchildren (8–11 years) belonging from five primary schools in the neighborhoods of Turin (northwest Italy) were enrolled and classified as Sedentary based on Triaxial Accelerometry and Physical Activity Questionnaire for Older Children (PAQ-C-It) (42).

Parents/guardians and teachers furnished written informed consent for participation in the study, adhering to the ethical standards outlined in the 1964 Declaration of Helsinki. The ethics committees on human research of the University of Turin (9 March 2020: Protocol #134691) and Naples (17 January 2020: Protocol #376/19) granted approval for the study.

Anthropometric measurements were reported. The procedures used to take anthropometric measures were standardized as described in Lupo et al. (42): height was measured using a portable stadiometer (Model 214; Seca, Hamburg, Germany), weight was measured using an electronic scale (Model 876; Seca, Hamburg, Germany), and waist circumference was measured while standing, halfway between the lowest rib and the iliac crest, using an Ana elastic meter. Body Mass Index (BMI) was calculated by dividing weight by height squared (kg/m^2^; Table 1). On average, the BMI of all enrolled schoolchildren fall within a range between the 75th and 85th percentile (43). For the daily dietary intake assessment all participants completed the questionnaire (3-Days food records). Records were processed using Winfood software (Medimatica S.u.r.l., Colonnella, TE, Italy). Statistical analysis was performed through Jamovi (2.3.26 version).

**Table 1:** Anthropometric characteristics of enrolled schoolchildren in Structured (Sa), Daily Mile (Dm) activities and Control (Ct) group, PRE-e POST-training intervention.

		Ct	Dm	Sa		Tukey *p*-value
Gender M/F		14 / 5	12 / 8	13 / 9	ANOVA *p*-value	
Age (years)		8–10	8–11	9–11		
Height (cm)	PRE	133.0 ± 5.3	134.0 ± 5.1	141.0 ± 6.9	0.934	Ct vs. Dm *p* = 0.48 Ct vs. Sa *p* < 0.01 Dm vs. Sa *p* < 0.01
	POST	139.0 ± 5.3	140.0 ± 5.9	147.0 ± 7.2		Ct vs. Dm *p* = 0.72 Ct vs. Sa *p* < 0.01 Dm vs. Sa *p* < 0.01
Weight (kg)	PRE	33.8 ± 8.0	33.4 ± 7.2	36.3 ± 7.6	0.610	Ct vs. Dm *p* = 0.99 Ct vs. Sa *p* = 0.44 Dm vs. Sa *p* = 0.38 Ct vs. Dm *p* = 0.99 Ct vs. Sa *p* = 0.33 Dm vs. Sa *p* = 0.37
	POST	37.6 ± 9.8	38.0 ± 10.7	42.4 ± 11.3		
BMI (kg/m^2^)	PRE	19.0 ± 3.5	18.5 ± 3.1	18.2 ± 2.8	0.664	Ct vs. Dm *p* = 0.89 Ct vs. Sa *p* = 0.84 Dm vs. Sa *p* = 0.95 Ct vs. Dm *p* = 0.97 Ct vs. Sa *p* = 1.00 Dm vs. Sa *p* = 0.97
	POST	19.3 ± 4.2	19.0 ± 4.2	19.3 ± 4.4		
Waist/Height (cm)	PRE	0.5 ± 0.1	0.5 ± 0.1	0.4 ± 0.1	0.764	Ct vs. Dm *p* = 0.71 Ct vs. Sa *p* = 0.13 Dm vs. Sa *p* = 0.50 Ct vs. Dm *p* = 0.68 Ct vs. Sa *p* = 0.09 Dm vs. Sa *p* = 0.36
	POST	0.5 ± 0.1	0.5 ± 0.1	0.4 ± 0.1		

*p*-values of two-way ANOVA and *post-hoc* Tukey comparisons were reported. BMI, Body Mass Index.

### 2.2 Training intervention

All sedentary enrolled schoolchildren were randomly assigned to 2 different types of exercise: Structured Exercise (Sa), 2 h/w muscle activation, strength and coordination exercises (supervised by a kinesiologist); Daily Mile (Dm), 1 h/w Sa plus 15 min/4 for week of walking/running outdoors (supervised by class teacher) or control group (curricular activities, 2 h/w PA supervised by class teacher, Ct) for 6-months (October, 2021–March, 2022). All interventions were planned within the common weekly volume of physical activity (i.e., 2 h), despite differently distributed according to the type of intervention. All children belonging to a single school class were enrolled in one of the three sub-groups, and no information about results of previous fitness measures (classification in active and no-active children) have been shared with them.

In particular, 22 schoolchildren belonging to the same class were involved in the Sa group and performed the following protocol: two (1 h in a day, and one in another day) school weekly hours of physical activity leaded by an expert (i.e., Sport Sciences Master degreed) according to *American College of Sports Medicine* guidelines (44). In particular, a single hour of this school physical activity type was distributed in 3 parts, 20 min/each focused on: fundamental movement skills, strength activities (strengthening activities) and aerobic activity. For the first part of exercises, basic body movements such as running, jumping, catching, balance, agility and coordination were proposed (45). The strength activities included all those movements that are generated by muscle contraction against resistance, aiming not only to improve individual strength level, but also anaerobic endurance and muscle size, balance, flexibility, mobility, posture, bone density, strength, self-esteem, prevention of chronic conditions such as diabetes, heart disease and depression, reduction in injury risk and pain management (46). Finally, for the last 20 min of the session, the proposed exercises were characterized by the contraction of large muscle groups, exclusively based on the aerobic energy system, by practicing walking, running, playing games, dancing, all activities recognized as able to increase cardiorespiratory fitness, weight maintenance, strengthens muscles and bones, mental health, social skills, and academic performance (44, 47). The second semi-structured intervention group (Dm) included a class of 22 schoolchildren that performed the following protocol: four weekly days characterized by the practice of *The Daily Mile*,^1^ plus the 1 h of curricular physical activity leaded by the generic teacher. In particular, for *The Daily Mile*, children used to go outside within the spaces belonging to the school, to jog and/or run independently for 15 min, supervised by the generic teacher. A class of 20 schoolchildren continued to carry out the curricular physical activities under the supervision of their generic teacher, control sub-group (Ct).

### 2.3 Physical fitness test

*Lower-Limb Muscle Power*: The standing long-jump test was performed to assess lower-limb power. The test was performed as previously reported (48). Two trials were performed (with a 1 min pause in between), and the best score was considered for statistical analysis. Not all enrolled children carried out the POST-intervention assessment, therefore the final sample size for this test was: Ct = 19, Dm = 21, Sa = 13.

*Balance*: Balance was tested using the single-leg stance test. The dominant leg of the participants was determined by asking them which was their favorite leg (49). The test was performed as previously reported (48). The time (in seconds) was recorded using a stopwatch with an accuracy of 0.01 s. Not all enrolled children carried out the POST-intervention assessment, therefore the final sample size for this test was: Ct = 19, Dm = 18, Sa = 21.

*Cardiorespiratory Fitness*: The 20 m shuttle-run test, according to the Leger test soundtrack version (CAEP Quebec Faca), was used to evaluate the cardiorespiratory fitness. The test was performed as previously reported (50). The test score was the last reached stage (converted into the corresponding distance expressed in meters) (51). Not all enrolled children carried out the POST-intervention assessment, therefore the final sample size for this test was: Ct = 19, Dm = 21, Sa = 21.

*Sprint Ability:* Children’s sprint ability was evaluated by the 20 m linear sprint test (52). Individually, participants started the test with feet behind the startling line (53) and ran the 20 m distance as fast as possible, twice with a 1 min rest between the trials. The time elapsed from the start to the finish line was measured through infrared reflex photoelectric cells (Witty—Wireless Training Timer; Microgate, Udine, Italy), measuring to the nearest 0.01 s. Not all children carried out the POST-intervention assessment, therefore the final sample size for this test was: Ct = 19, Dm = 22, Sa = 21.

### 2.4 Saliva sample collection and genomic DNA extraction

The volunteers were instructed not to consume food and refrain from using mouth cleanliness products 1 h prior to saliva retrieval. At minimum, 2 milliliters of unstimulated spit were gathered, chilled, and preserved at −80°C until analysis. DNA extraction from saliva specimens was accomplished utilizing the MagPurix Bacterial DNA Extraction Kit (ZP02006; Zinexts Life Science Corp.) following the manufacturer’s instructions. DNA concentration was measured utilizing the Qubit dsDNA BR and HS assay kit (Life Technologies, CA, United States).

### 2.5 Preparation of the 16 S rRNA sequencing library (FIX)

PCR amplification was conducted to target the variable V3-V4 segments of the 16S rRNA bacterial genes. Specific primers embedded with barcodes and potent enzymes were utilized for PCR execution. The PCR primer sequences were as follows: (forward 341F: CCTAYGGGRBGCASCAG; reverse 806R: GGACTACNNGGGTATCTAAT). PCR products ranging from 450 to 500 base pairs were isolated using 2% agarose gel electrophoresis. For library construction, equimolar amounts of PCR products from each sample were combined, end-repaired, A-tailed, and subsequently ligated with Illumina adapters. Library quality control was conducted using Qubit and real-time PCR for quantification, and bioanalyzer for verifying the insert size distribution. Libraries were sequenced on a paired-end Illumina platform, producing 250 bp paired-end raw reads. The raw sequencing data are available in Zenodo (Publication date: July 1st, 2024).^2^

### 2.6 Bioinformatic analysis

We conducted the analyses as reported in Mancini et al. (41). In brief, we used the R package DADA2 and its workflow (54) to infer the Amplicon Sequence Variants (ASVs) and for the taxonomic assignments.

Briefly, we first filtered and trimmed raw sequencing reads to remove low quality bases, adapters and identical reads. Then, the reads were denoised, merged and filtered to remove artifacts (PCR, and PhiX related chimeras). Then, we obtained the ASVs counts and assigned taxonomy level annotations using the SILVA database (version: v138, nr99) (55). The data were structured in objects including the ASVs quantifications, the taxonomy annotations, the sample group information and the phylogenetic tree using the phyloseq and the APE packages (56, 57). Finally, based on the initial DNA concentration, we removed possible contaminant ASVs by using the “prevalence” method of the decontam package (58). For the downstream analyses we used the MicrobiomeAnalystR package (59, 60) and conducted data normalization, measures of diversity and differential abundance estimation. As previously described (61), we filtered and normalized the raw ASV counts based on their low abundance (at least the 20% of a given ASV values should contain at least 4 counts) and low variance (based on Inter-quantile range ± 10%) in order to discard possible sequencing errors. Moreover, ASVs showing constant values in all samples could be excluded from the comparative analyses. Finally, we used the total sum scaling in order to obtain normalized ASV counts. Based on these counts, we conducted the downstream analyses in order to evaluate the alpha-and beta-diversity reporting the species richness and evenness and the diversity of species between the groups. Then we evaluated the species richness from the results of sampling and whether samples were sufficiently sequenced to represent their identities by using the rarefaction analysis. Moreover, we measured the differential abundance with the LEfSe (Linear Discriminant Analysis Effect Size) algorithm (62) for biomarker discovery. Finally, we used PICRUSt (Phylogenetic Investigation of Communities by Reconstruction of Unobserved States) to infer the metabolic potential of microbial communities (KEGG pathways). In this analysis, we started from the ASVs belonging to the significant genera and species obtained by the differential abundance analysis (LefSE algorithm).

### 2.7 Statistics

We calculated the ACE and Chao1 indices (for accounting species richness) and the Fisher metrics (to consider both richness and evenness).

To evaluate the alpha-diversity, we calculated the ACE and Chao1 indices and the Fisher metrics and assessed the statistical significance of group comparisons between the groups of samples under study by using the Mann–Whitney test. We evaluated diversity of species between the groups (the beta-diversity) by PCoA (Principal Coordinates Analysis) of the UniFrac distances and assessed the statistical significance with the PERMANOVA test. The statistical significance of rarefaction profiles was assessed by using the Mann–Whitney test. We assessed the statistical significance by using the Mann–Whitney test. Finally, we measured the differential abundance with the LEfSe algorithm (62) for biomarker discovery. It involves the Kruskal-Wallis rank sum test to identify features (e.g. Species or Genera) with significant differential abundance in the groups, followed by linear discriminant analysis (LDA) to evaluate the relevance (the effect size) of the selected features. Differentially abundant features were considered if the *p*-value was less than or equal to 0.05 and if the Log LDA was greater than or less than 0.5. We used PICRUSt to infer the metabolic potential of microbial communities (KEGG pathways). In this analysis, we started from the ASVs belonging to the significant genera and species obtained by the differential abundance analysis (LefSE algorithm). Statistical analysis of variables shown in Tables 1–3 were performed using Jamovi software (version 2.2.5.0). The normality of the data was verified by applying the Shapiro–Wilk test and mean ± standard deviation (SD) for each variable reported in Tables. Comparisons between groups were determined with Two- or One-way ANOVA for all normally distributed variables or with Kruskal-Wallis H test for non-parametric variables. Paired Sample t-test within intervention groups for physical fitness parameters was performed and followed by Effect size (>0.2 and < 0.5 small; >0.5 and < 0.8 moderate; >0.8 large). The level of significance was set at *p* < 0.05.

## 3 Results

### 3.1 Cohort characteristics

Anthropometric measurements of the schoolchildren participating in this study, were acquired before and after the training intervention (PRE-POST-) and reported in Table 1. No significant difference in the gender frequency of individuals was found [Χ^2^ (2) = 1.14, *p* = 0.57]. Basal differences (PRE-) found in height [F (2, 63) = 19.4; *p* < 0.001 one-way ANOVA followed by Tukey *post-hoc* test revealed a statistically significant difference (*p* < 0.01) in Ct vs. Sa and Dm vs. Sa] can be attributed to the school class to which the children belong. Weight, BMI, Waist/Height parameters did not vary among groups in baseline conditions (Krustal-Wallis One-way ANOVA; *p* > 0.05). Furthermore, two-way ANOVA revealed that there was not a statistically significant interaction between time (PRE-and POST-) and anthropometric measurements (relatives *p*-value and Tukey *post-hoc* were reported in Table 1).

On the same time, One-way ANOVA revealed no significant differences in all diet components analyzed among Sa, Dm and Ct groups (Table 2).

**Table 2:** Eating habits of enrolled schoolchildren in Structured (Sa), Daily Mile (Dm) activities and Control (Ct) group.

	Ct	Dm	Sa	*p*-value
Gender M/F	14 / 5	12 / 8	13 / 9	
Calories (kcal)	1559.0 ± 172.0	1413.0 ± 188.0	1502.0 ± 320.0	0.060
Carbohydrates (%)	47.6 ± 3.0	48.0 ± 6.5	46.9 ± 2.8	0.652
Lipids (%)	35.8 ± 2.8	37.0 ± 4.6	36.7 ± 2.2	0.525
Saturated/fatty acids (%)	37.9 ± 6.0	38.0 ± 6.9	36.5 ± 5.9	0.669
Proteins (%)	15.7 ± 1.5	15.1 ± 2.8	16.1 ± 1.6	0.429
Animal proteins/Proteins (%)	75.3 ± 9.4	67.6 ± 18.7	76.6 ± 7.6	0.293
Vegetal proteins/Proteins (%)	24.8 ± 9.4	32.4 ± 18.7	23.4 ± 7.6	0.293
Total fiber/1000 kcal (%)	7.0 ± 1.7	7.0 ± 2.4	6.5 ± 1.6	0.667

### 3.2 Physical fitness test

The schoolchildren were assessed for physical fitness both before and after the 6-mo training intervention (PRE-and POST-). Significant PRE-POST improvements were observed for lower limb power (standing long-jump) and sprint (linear 20 m) only in Sa group. Conversely, balance (single-leg stance) and endurance (20 m shuttle-run test) resulted significantly improved in both Sa and Dm groups; slight significant improvement only in balance test was found in Ct (Table 3). Two-way ANOVA (Type of intervention × Time PRE/POST with Physical test as dependent variable) resulted not significant for 20 m shuttle-run, Single-leg stance and Sprint (*p* > 0.05). Differently, significant interaction between time (PRE-and POST-) and type of intervention for Standing long-jump test [F (2, 50) = 4.91; *p* = 0.011] was found.

**Table 3:** Physical fitness test of enrolled schoolchildren in Structured (Sa), Daily Mile (Dm) activities and Control (Ct) group, PRE-e POST-training intervention.

				
Test	Group	Pre	Post	*p*-value	Effect size
Standing long-jump (cm)	Ct	118 ± 19.8	122 ± 28.3	0.360	- 0.22
	Dm	109 ± 16.6	113 ± 23.0	0.091	- 0.39
	Sa	115 ± 18.2	139 ± 21.8	< 0.001	- 1.35
20 m shuttle-run (m)	Ct	512 ± 203	639 ± 397	0.074	- 0.44
	Dm	459 ± 313	583 ± 349	0.008	- 0.68
	Sa	585 ± 206	779 ± 268	0.004	- 0.71
Single-leg stance (s)	Ct	28.3 ± 27.5	46.2 ± 30.9	0.046	- 0.53
	Dm	25.3 ± 29.8	69.3 ± 51.9	0.008	- 0.74
	Sa	55.0 ± 34.9	93.3 ± 39.9	0.009	- 0.67
Sprint (s)	Ct	4.5 ± 0.4	4.3 ± 0.5	0.095	0.40
	Dm	4.4 ± 0.3	4.4 ± 0.4	0.673	0.09
	Sa	4.3 ± 0.3	3.9 ± 0.3	0.004	0.71

### 3.3 Sequencing reads processing and taxonomic assignments

The Illumina sequencing of the hypervariable V3-V4 regions of the 16S rRNA bacterial genes generated 2×250 bp paired-end reads. Overall, the percentage of bases with quality scores above 20 and 30 (Q20 and Q30, respectively) was of 97.47 and of 92.93, respectively (Supplementary Figure S1A). The percentage of GC nucleotides was of 51.85 (Supplementary Figure S1B).

On average, we obtained 172,066 reads per-sample (min = 150,914; max = 189,035). The raw reads were filtered and trimmed (median = 171,879; min = 150,769; max = 188,851), denoised on the forward (median = 170,557; min = 149,387; max = 187,291) and reverse (median = 170,671; min = 149,387; max = 187,291) directions, merged (median = 149,274; min = 112,706; max = 170,971) and chimeric reads removal (median = 114,432; min = 87,964; max = 136,831). After merging, the median length of reads was of 425 bp (min = 421; max = 427; Supplementary Figures S1C,D).

We assigned taxa using the SILVA database of non-redundant sequences (version: v138, nr99). Overall, we could identify a total of 4,759 taxa (ASVs) that were taxonomically assigned to the kingdom of bacteria. The 97.92% of the ASVs was annotated at the phylum level (*n* = 13 phyla), the 97.75% at the class level (*n* = 21 classes), the 97.18% at the order level (*n* = 56 orders), the 92.54% at the family level (*n* = 96 families), the 82.92% at the genus level (*n* = 215 genera) and the 6.51% was annotated up to the species level (*n* = 172 species; Supplementary Figure S1E). From the initial set of annotated ASVs, we discarded a total of 31 taxa as possible contaminants. Moreover, as described in Methods we removed low abundant and low variable ASVs to obtain the final set of 3,503 ASVs that was normalized and used for down-stream analyses.

### 3.4 Diversity estimates

**Figure 1:**
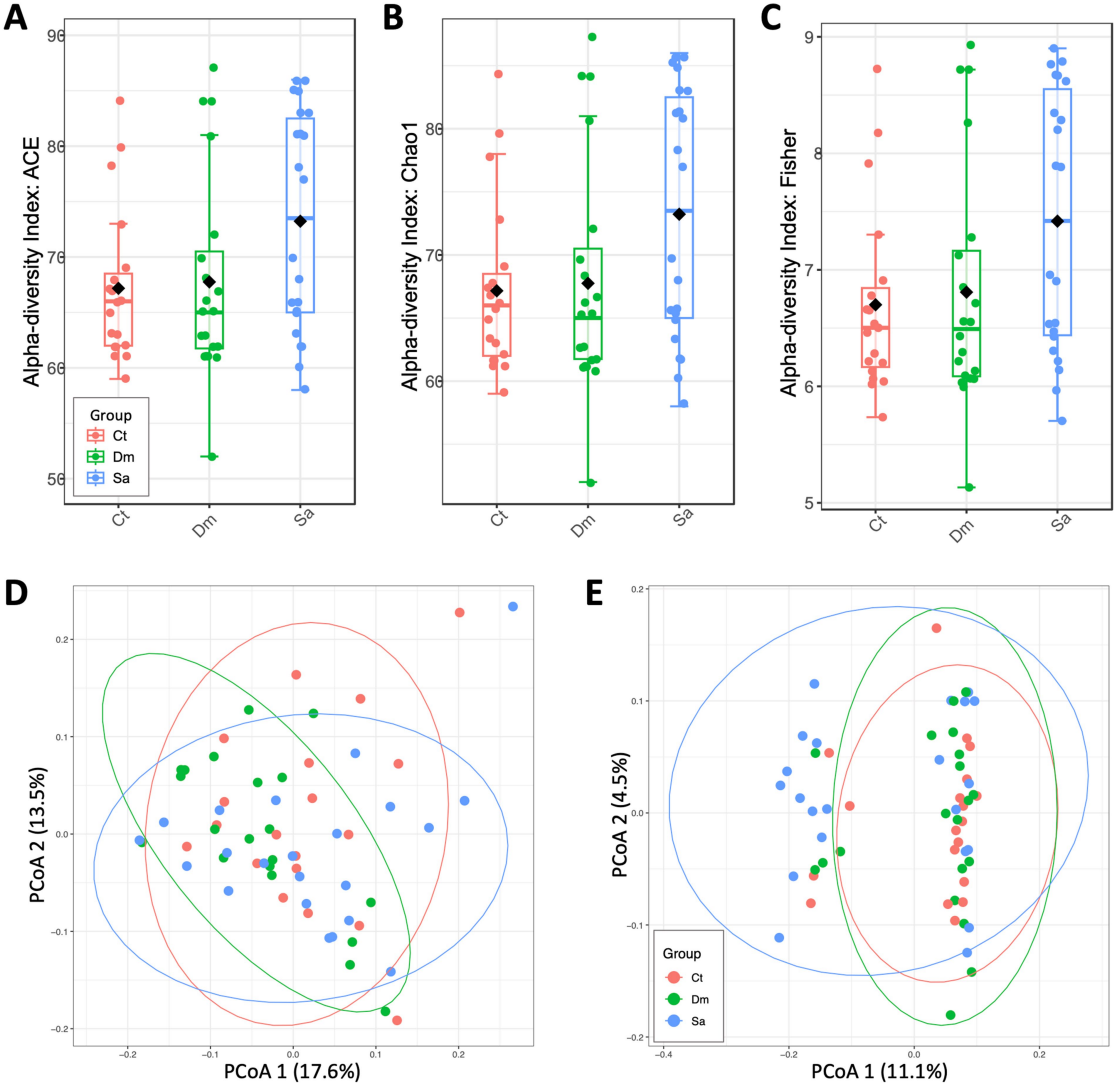
Genus-level diversity and distances measured between sedentary Controls (Ct), Daily Mile activity (Dm) and Structured activity (Sa). A| Alpha-diversity measured by ACE index (Ct vs. Dm: *p* = 0.955; Ct vs. Sa: *p* = 0.063; Dm vs. Sa: *p* = 0.106). B| Alpha-diversity measured by Chao1 index (Ct vs. Dm: *p* = 0.955; Ct vs. Sa: *p* = 0.061; Dm vs. Sa: *p* = 0.106). C| Alpha-diversity measured by Fisher index (Ct vs. Dm: *p* = 0.923; Ct vs. Sa: *p* = 0.048; Dm vs. Sa: *p* = 0.116). D| Principal Coordinates Analysis plot of beta-diversity index measured by weighted UniFrac distances (*p* = 0.147). E|Principal Coordinates Analysis plot of beta-diversity index measured by unweighted UniFrac distances (*p* = 0.024). A, B, C: Mann–Whitney test. D, E: PERMANOVA test.

We estimated the alpha-diversity at the genus level. Overall, we observed slight, no significant differences among the groups (Ct, *n* = 19; Dm, *n* = 20; Sa, *n* = 22) indeed, ACE (Figure 1A), Chao1 (Figure 1B) indexes are not significant, *p* > 0.05 (Mann–Whitney test). In particular, the SA group showed higher richness and evenness (Fisher metrics; Figure 1C) compared to the Control Group (Ct, *p* = 0.048).

The beta-diversity analysis, evaluated by weighted UniFrac distance metric did not evidentiate significant differences in microbial composition among the groups (R^2^ = 0.0414, *p* = 0.147, PERMANOVA test; Figure 1D). Differently, the microbial composition resulted significant different when measured by unweighted UniFrac distances (R^2^ = 0.04229, *p* = 0.024, PERMANOVA test; Figure 1E). Moreover, the rarefaction analysis clearly showed that we were able to capture in depth the species richness from the results of sampling and sequencing in the groups without any statistically significant difference between the groups under study (*p* > 0.8; Mann–Whitney test) Indeed, we can assess that the variation observed between the two groups was due to the different taxa abundance and to the types of taxa present in their saliva microbiome.

### 3.5 Abundance estimates

We evaluated and compared the taxa abundance in the final set of 3,503 filtered and normalized ASVs (see above). Overall, we identified *n* = 13 phyla, *n* = 21 classes, *n* = 56 orders, *n* = 96 families, *n* = 215 genera and *n* = 172 species.

**Figure 2:**
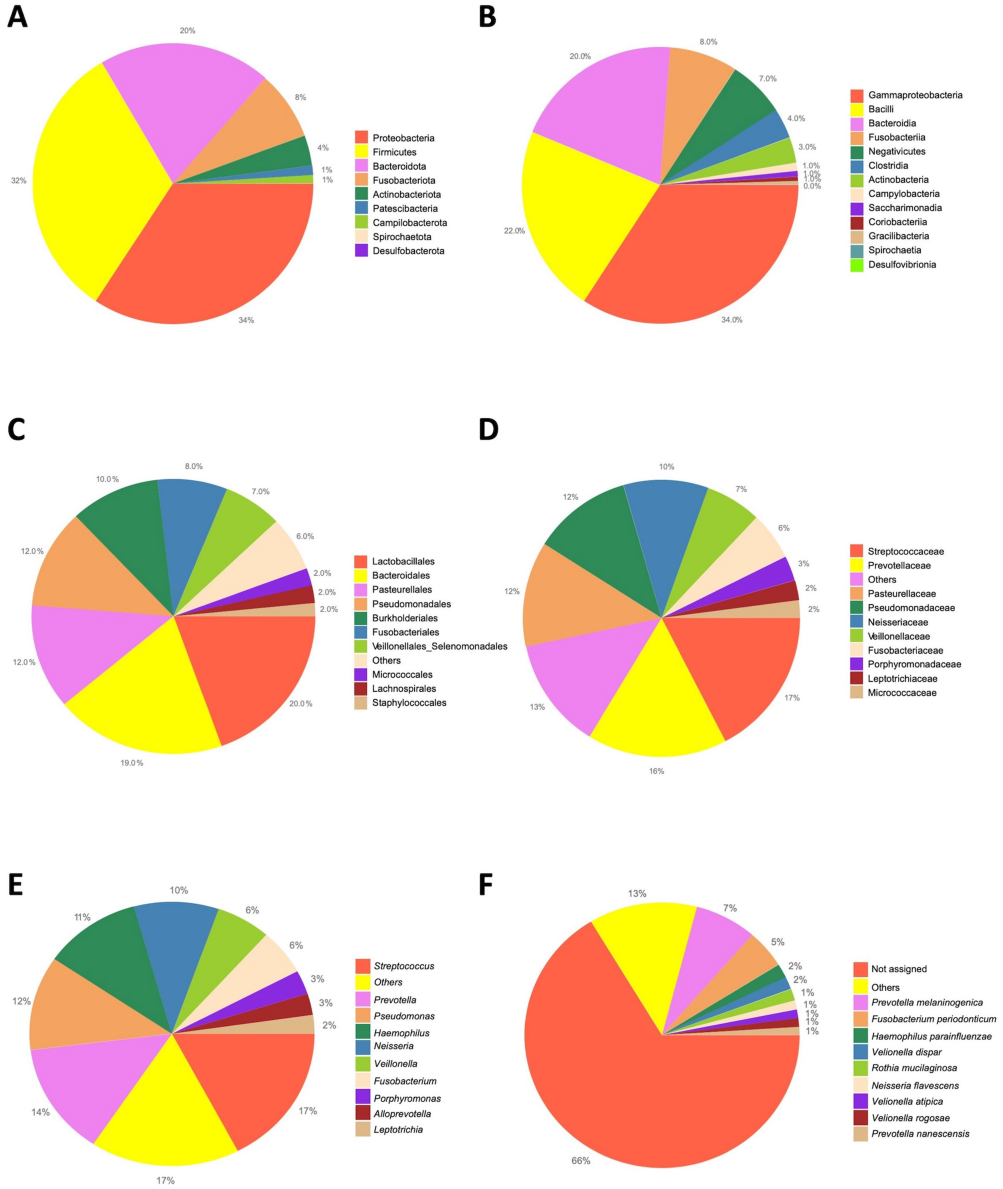
Overall taxonomic abundance. The pie charts report the overall abundance of the identified taxa. A| Phylum level. B| Class level. C| Order level. D| Family level. E| Genus level. F| Species level.

At the phylum level, on average, the most abundant bacteria were the Proteobacteria, the Firmicutes, the Bacteroidota accounting for the 34.27, 32.27%, and the 19.93%, respectively (Figure 2A). The most represented classes were the Gammaproteobacteria (34.27%), the Bacilli (21.99%), and the Bacteroidia (19.93%; Figure 2B). The most prevalent orders were the Lactobacillales (19.52%), the Bacteroidales (19.45%), the Pasteurellales (12.3%) and the Pseudomonadales (11.72%; Figure 2C). Among the most abundant families, Streptococcaceae (17.37%), the Prevotellaceae (16.35%), the Pasteurellaceae (12.3%) and the Pseudomonadaceae (11.55%; Figure 2D) were found. At the genera level, the *Streptococcus* (17.36%), *Prevotella* (13.72%), *Pseudomonas* (11.55%), *Haemophilus* (11.13%), and *Neisseria* (9.63%; Figure 2E) are the most aboundant. Finally, the top abundant species that we were able to classify were the *Prevotella melaninogenica* (7.28%), *Fusobacterium periodonticum* (4.69%), *Haemophilus parainfluenzae* (1.73%), *Veillonella dispar* (1.53%) and the *Rothia mucilaginosa* (1.46%; Figure 2F).

Taken together, the diversity and the overall abundance estimates are in line with our recent results (41). Indeed, we can assess that the data resulting from the sampling, sequencing and analyses protocols are reliable. Moreover, the variation observed among the groups, although slight and probably due to the small sample size, is due to the different taxa abundances and to the types of taxa in the analyzed microbiomes.

### 3.6 Differential abundance estimates

**Figure 3:**
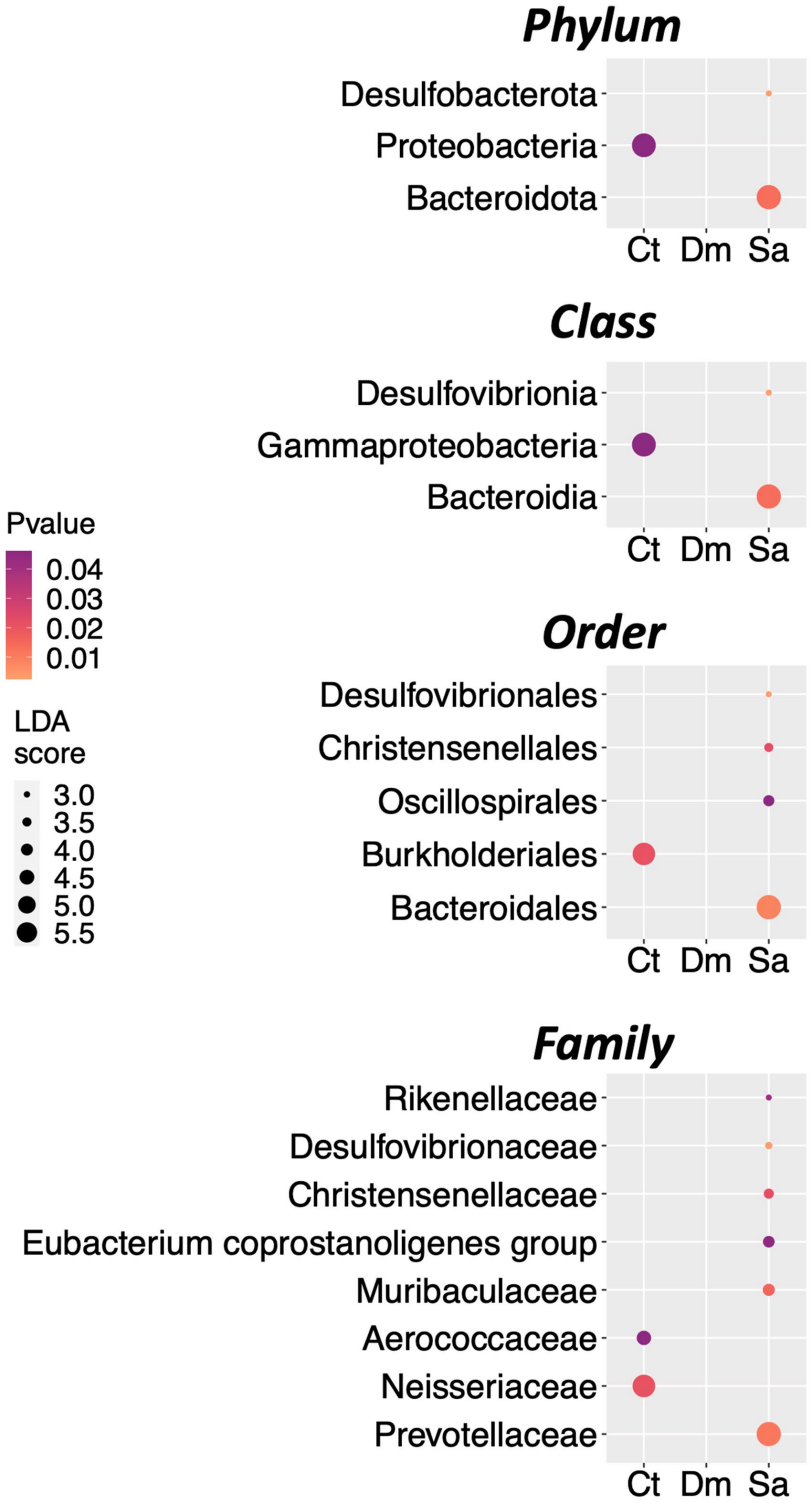
Differentially abundant taxa. The results of LEfSe analysis, are reported. The dots sizes are proportional to the score of the LDA algorithm whereas the dot graduation color is proportional to the significance level (Kruskal-Wallis rank sum test). From top to bottom: Dot plots showing the differentially abundant Phyla, Classes, Orders, and Families.

As described in Methods we used the LEfSe algorithm to perform the differential abundance analysis and to find the taxa that could explain the differences between the groups under study. We set the significance threshold at 5% and the Log LDA cutoffs at ±0.5. With these stringent filters, we observed the first significant differences at the Phylum level. Indeed, the Bacteroidota (LDA score = 5.61; *p* = 0.013) and the Desulfobacterota (LDA score = 2.8; *p* = 0.002) were more abundant in the Sa group as compared to the others. Conversely, the Phylum of the Proteobacteria was enriched in the Ct group (LDA score = 5.54; *p* = 0.042; Figure 3). At the Class level, the Bacteroidia (LDA score = 5.61; *p* = 0.013) and the Desulfovibrionia (LDA score = 2.8; *p* = 0.003) were more abundant in the Sa group whereas the Gammaproteobacteria was enriched in the Ct group (LDA score = 5.54; *p* = 0.042; Figure 3). In details, five Orders (four in the Sa and one in the Ct group, respectively) and eight Families (six in the Sa and two in the Ct group, respectively) were differentially abundant after the LEfSe analysis (Figure 3).

**Figure 4:**
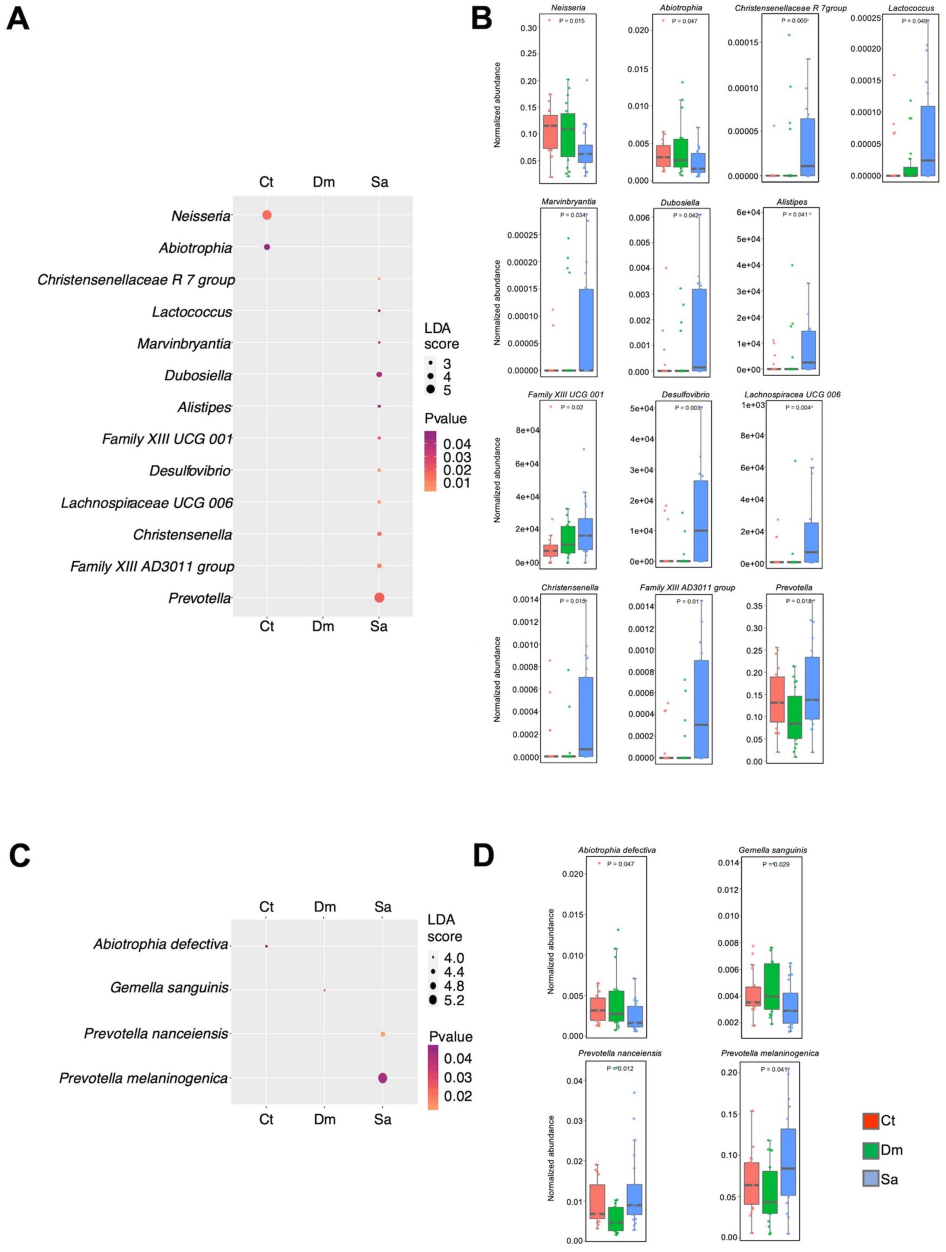
Differentially abundant taxa. The results of LEfSe analysis, are reported. The dots sizes are proportional to the score of the LDA algorithm whereas the dot graduation color is proportional to the significance level (Kruskal-Wallis rank sum test). A| Dot plot showing the differentially abundant genera. B| Box plots showing the normalized abundance levels of genera reported in panel A. C| Dot plot reporting the differentially abundant species. D| Box plots showing the normalized abundance levels of genera reported in panel C. Ct, sedentary controls; Dm, Daily Mile activity; Sa, Structured activity.

Furthermore, we observed that 13 genera were responsible for the differences between the groups (Figures 4A,B). In particular, the *Prevotella* (LDA score = 5.56; *p* = 0.018), the *Dubosiella* (LDA score = 3.68; *p* = 0.042) and the *Family XIII AD3011 group* (LDA score = 3.28; *p* = 0.010) were the most abundant genera in the Sa group. Conversely, the *Neisseria* (LDA score = 5.34; *p* = 0.014) and the *Abiotrophia* (LDA score = 3.98; *p* = 0.047) were more abundant in the Ct group.

Four species showed significant results in the LEfSe analysis (Figures 4C,D). Indeed, the *Prevotella melaninogenica* (LDA score = 5.3; *p* = 0.041) and the *Prevotella nanceiensis* (LDA score = 4.4; *p* = 0.012) were more abundant in the Sa group. The *Abiotrophia defectiva* (LDA score = 3.98; *p* = 0.047) was enriched in the Ct group. Of note, the *Gemella sanguinis* (LDA score = 3.92; *p* = 0.029) was the only enriched species in the Dm group.

### 3.7 Metabolic pathways reconstruction

**Figure 5:**
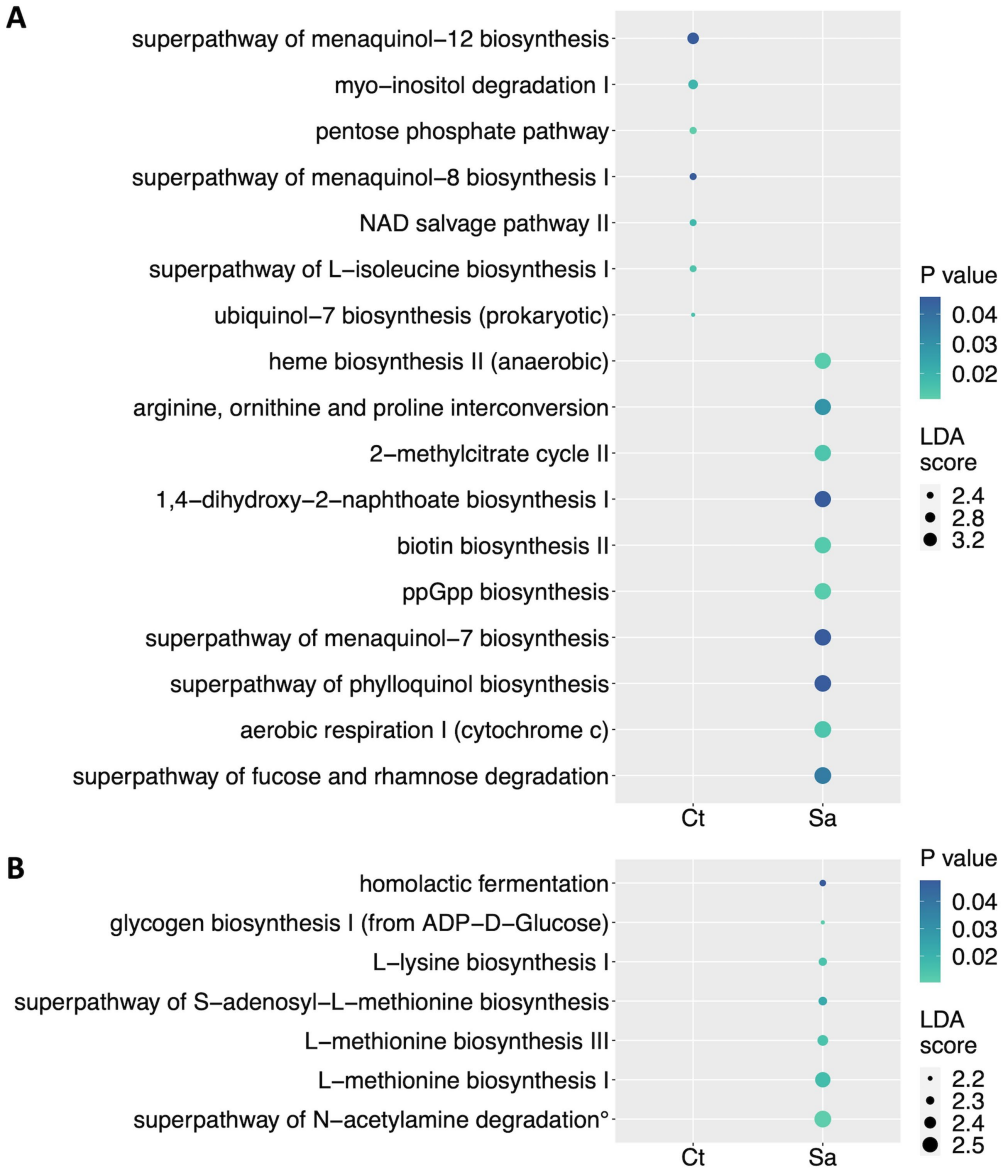
Metabolic pathways reconstruction. PICRUSt (Phylogenetic Investigation of Communities by Reconstruction of Unobserved States) was used to predict the activity of metabolic pathways (KEGG) starting from the significant taxa obtained by LefSE analysis. A| Dot plot showing the differentially represented pathways at the genus level. B| Dot plot showing the differentially represented pathways at the species level. Ct, sedentary controls; Sa, Structured activity. (°) superpathway of N-acetylglucosamine, N-acetylmannosamine and N-acetylneuraminate degradation. In this figure the dots sizes are proportional to the score of the LDA algorithm whereas the dot graduation color is proportional to the significance level (Mann–Whitney test).

We used PICRUSt (Phylogenetic Investigation of Communities by Reconstruction of Unobserved States) to predict the activity of metabolic pathways (KEGG) starting from the significant genera and species obtained by LefSE analysis between the Ct and Sa groups. At the genus level, PICRUSt analysis highlighted the prevalence of the superpathway of fucose and rhamnose degradation (LDA score = 3.59; *p* = 0.037), the aerobic respiration I (cytochrome c; LDA score = 3.59; *p* = 0.014), the superpathway of phylloquinol biosynthesis (LDA score = 3.57; *p* = 0.046), the superpathway of menaquinol-7 biosynthesis (LDA score = 3.55; *p* = 0.046), the ppGpp biosynthesis (LDA score = 3.55; *p* = 0.012), the biotin biosynthesis II (LDA score = 3.54; *p* = 0.013), the 1,4-dihydroxy-2-naphthoate biosynthesis I (LDA score = 3.53; *p* = 0.046), the 2-methylcitrate cycle II (LDA score = 3.53; *p* = 0.014), the arginine, ornithine and proline interconversion (LDA score = 3.52; *p* = 0.029), the anaerobic heme biosynthesis II (LDA score = 3.52; *p* = 0.012) for the Sa compared to the Ct group. By contrast, the Ct group showed an enrichment of the ubiquinol-7 biosynthesis (LDA score = 2.06; *p* = 0.016), the superpathway of L-isoleucine biosynthesis I (LDA score = 2.4; *p* = 0.014), the NAD salvage pathway II (LDA score = 2.4; *p* = 0.018), the superpathway of menaquinol-8 biosynthesis I (LDA score = 2.41; *p* = 0.046), the pentose phosphate pathway (LDA score = 2.43; *p* = 0.011), the myo-inositol degradation I (LDA score = 2.73; *p* = 0.02), the superpathway of menaquinol-12 biosynthesis (LDA score = 2.96; *p* = 0.046; Figure 5A).

At the species level, we found that only the Sa showed enriched pathways, compared to Ct group. Indeed, we found enrichment of the superpathway of N-acetylglucosamine, N-acetylmannosamine and N-acetylneuraminate degradation (LDA score = 2.54; *p* = 0.011), the L-methionine biosynthesis I (LDA score = 2.5; *p* = 0.017), the L-methionine biosynthesis III (LDA score = 2.37; *p* = 0.014), the superpathway of S-adenosyl-L-methionine biosynthesis (LDA score = 2.31; *p* = 0.023), the L-lysine biosynthesis I (LDA score = 2.3; *p* = 0.014), the homolactic fermentation (LDA score = 2.25; *p* = 0.048), the glycogen biosynthesis I (from ADP-D-Glucose; LDA score = 2.18; *p* = 0.011; Figure 5B).

## 4 Discussion

In this study, we assessed the effects of 6-mo of Sa or Dm exercise on physical fitness and saliva microbiota composition in sedentary schoolchildren aged 8 to 11.

The following highlights the most relevant results discussed:

- After the intervention, the Sa group showed enhancements in lower limb power and sprint ability, while both the Sa and Dm groups showed improvements in endurance and balance. Instead, the Ct group showed a slight improvement only in the balance test;
- Among the genera differentially enriched in saliva after the training intervention, *Prevotella*, *Dubosiella*, and the *Family XIII AD3011* group were the most abundant in the Sa group. Conversely, *Neisseria* and *Abiotrophia* were prevalent in the Ct group;
- Four species showed significant differences: *Prevotella melaninogenica* and *Prevotella nanceiensis* most abundant in the Sa group, *Gemella sanguinis*, enriched in the Dm group and *Abiotrophia defectiva* in the Ct group’s saliva, respectively.

It has been demonstrated that the Gut microbiota (GM) composition affects different physiological functions of the host. Many factors influence the GM composition, including diet and exercise, and alterations in GM composition have been associated with health or different chronic diseases such as diabetes and obesity in humans (3, 64–66). Recent evidence indicates that exercise affects gut microbiota composition by increasing the *Bacteroidetes*/*Firmicutes* ratio in GM (67, 68). Training duration, type (aerobic vs. resistance) and intensity also affects GM composition. A reduction in the *Firmicutes*/*Bacterioides* ratio with an increase in the *Bacteroides* was observed in high-intensity and moderate-intensity PA (69, 70), with beneficial bacteria enrichment in aerobic exercise (71, 72). In athletes practicing intense and prolonged exercise, the effects on GM composition seem controversial, showing higher richness in beta-diversity in athletes compared to controls, with specific enrichments in *Firmicutes* and *Firmicutes prausnitzii*, which produce butyrate associated to intestinal health (1, 73, 74). On the other hand, there is an increase in bacteria involved in inflammatory processes, as evidenced by Roberts and colleagues (75). Conversely, moderate exercise seems to improve gut microbiota composition and intestinal barrier (76). Greater microbial diversity was also associated with cardiorespiratory fitness in healthier subjects (77). Furthermore, the host’s age plays an important role in GM composition mediated by exercise.

Exercise undertaken during early life increase the presence of *Bacteroidetes* associated with a lean phenotype and reduce *Firmicutes* associated with obesity (78, 79). It seems that the lower species richness found in the GM of young rats (80) and in humans (81) can be more easily modified by environmental factors, including exercise, compared to adults.

Saliva microbiota composition was closely associated with GM. We previously identified eight species more abundant in the saliva of Active compared to Sedentary schoolchildren, including *Prevotella nigrescens*, *Colinsella aerofaciens*, *Simonsella muelleri*, and *Parabacterioides merdae*. The prevalence of *Parabacteriodes* in the Active group also resulted in the activation of superpathway involved in carbohydrates metabolism and metabolites secretion, including SCFAs which are associated to healthier profile and reduced risk for dysmetabolic diseases (41).

Recently, multicentre EU projects (IDEFICS ‘Identification and prevention of Dietary and lifestyle-induced health EFfects in Children and infantS’ and HELENA ‘HEalthy Lifestyle in Europe by Nutrition in Adolescence’) (51, 82) have provided a large database for fitness reference values for children and adolescents from different European countries. Very recently, building on these studies, the FitBack European network highlighted the main health-related fitness components: cardiorespiratory fitness (20 m shuttle run test), muscular strength (handgrip strength and standing long jump tests), anthropometric measures (such as BMI, waist circumference, etc.) for children and adolescents aged 6–18 years (83).

Here we focused on the effects mediated by 6-mo of different exercise type (Structured vs. semi-structured) versus curricular on saliva microbiota composition in the Sedentary schoolchildren.

Interestingly, 6-mo structured (Sa) and semi-structured (Dm) exercise improved physical fitness markers (muscular power, cardiovascular fitness and balance) associated to the health together with an enrichment in saliva species abundance compared to Curricular exercise (Ct). The most abundant species found in saliva were *Prevotella melaninogenica* and *Prevotella nanceiensis* in Sa, *Gemella sanguinis* in Dm, and *Abiotrophia defective* in the Ct group.

Raju et al. recently analyzed the composition of saliva microbiota in a large cohort of Danish children, highlighting genera such as *Prevotella*, *Veillonella*, *Streptococcus*, *Selenomonas*, *Neisseria*, and *Gemella* as most abundant in saliva. Furthermore, the authors pointed-out the association between the abundance of *Prevotella* genera and body weight in children, suggesting that an enrichment of *Prevotella* in saliva could potentially serve as new marker to identify overweight risk in children (84). Previously, we also reported an increase in genera such as *Gemella*, *Prevotella*, *Streptococcus*, *Heamophilus*, *Neisseria* and *Veillonella* in the saliva of Italian schoolchildren. We observed an enrichment in species such as *Prevotella pallens* and *Neisseria mucosa* in Sedentary children, with a slight increasing trend in BMI index compared to Active group in line with the findings of Raju et al. (41, 84).

*Prevotella* is one of the most abundant genera in different human sites such as skin, oral cavity, gastrointestinal tract and is dominant in the oral cavity (85). Lifestyle factors, including exercise, diet, age and gender affect the expression of different species of *Prevotella* in body sites. *Prevotella* prevalence in saliva accounts for approximately 13% of the entire oral microbiota (85) in healthy subjects and around 10% in school-aged population in non-Westernized regions (86). *Prevotella melaninogenica* and *Prevotella nanceiensis* represent the most abundant species in the oral cavity of not-Westernized population, including Italian (86). The role of *Prevotella* in health and disease is not yet fully understood, positive association have been found with lower BMI (87, 88), although *Prevotella* is also involved in biofilm formation associated with poor oral hygiene and oral diseases (89). Diet composition also influences the expression of gut *Prevotella*, with enrichment in gut microbiota potentially promoting healthier lipid and glucose metabolism and aiding weight-loss (87, 88, 90–93).

Our results demonstrated that the structured physical activity intervention causes a shift in sedentary children from species associated with disease, such as *Neisseria mucosa*, to species associated with a healthier profile, such as *Prevotella melaninogenica*. This is in line with literature and suggests the effectiveness of the structured exercise program, supervised by Kinesiologist in improving the saliva microbiota related to a healthier profile. Interestingly, this effect was independent from the food habits of schoolchildren.

In addition, the PICRUSt analysis revealed a significant prevalence of several metabolic pathways in the Sa group compared to the Ct group. Remarkable pathways included the superpathway of fucose and rhamnose degradation and ppGpp biosynthesis. Both the degradation of oligosaccharides such as fucose and rhamnose and the biosynthesis of ppGpp promote the establishment of a healthy microbiota and protect against infection (94, 95). Furthermore, the superpathway of phylloquinol biosynthesis and menaquinol-7 biosynthesis were significantly highlighted. Phylloquinone, or vitamin K1 and menaquinone-7, or vitamin K2, are the main forms of vitamin K. Vitamin K has recently been attributed with antioxidant and anti-inflammatory properties and is involved in crucial events associated with aging (96). A recent meta-analysis combining data from three large cohorts reported that low circulating phylloquinone concentrations are associated with an increased risk of all-cause mortality (97). Meanwhile, other studies suggest that high vitamin K2 intake may help prevent cardiovascular disease (CVD) (98).

Finally, the interconversion of arginine, ornithine, and proline, as well as L-methionine biosynthesis I and III, were significantly prevalent in the Sa compared to the Ct group. Arginine, ornithine, and methionine are responsible for the synthesis of polyamines, small aliphatic polycations that play crucial roles in different cellular mechanisms. Additionally, polyamines help protect against oxidative stress by regulating proteins involved in the oxidative stress response and acting as ROS scavengers at physiological pH (99). These findings suggest that structured exercise induces significant metabolic adaptations that could be beneficial for health.

### 4.1 Strengths and limitations

Our study presents several strengths: the design and implementation of a structured and controlled training model in primary schoolchildren allowed us to obtain information, for the first time, on the effect of this type of exercise on the salivary microbiota in children. The homogeneity of age-matched schoolchildren belonging from the same geographical area, with similar eating habits, do not assuming drugs in days prior the analysis and with a good oral hygiene, enabled us to highlight the effect of physical exercise on the composition of the salivary microbiota, minimizing the influence of confounding variables (63, 81, 100).

However, this study presents some limitations: first, the limited number of children tested. Due to the small sample size, we were unable to perform a gender-specific analysis of saliva microbiota composition, which may introduce bias, as the extent to which gender influences the diversity and abundance of the salivary microbiota remains unclear (84, 101). We aim in future studies, to increase the sample size and provide a gender-based analysis of saliva microbiota composition.

## 5 Conclusion

In conclusion, our study highlights the significant impact of a structured exercise regimen on improvements in health-associated physical fitness markers and the abundance of beneficial species in the saliva microbiota in sedentary schoolchildren, contributing to the growing body of evidence supporting integrating structured exercise programs into school curricula to promote health in children.

## Data Availability

The datasets presented in this study can be found in online repositories. The names of the repository/repositories and accession number(s) can be found at: https://doi.org/10.5281/zenodo.12607061, 10.5281/zenodo.12607061.

## Footnotes

^1^ http://thedailymile.co.uk/

^2^ https://doi.org/10.5281/zenodo.12607061

## References

[1] Turnbaugh PJ Ley RE Hamady M Fraser-Liggett CM Knight R Gordon JI. The human microbiome project. Nature. (2007) 449:804–10. doi: 10.1038/nature06244, PMID: 17943116 PMC3709439

[2] Pérez-Prieto I Migueles JH Molina NM Sola-Leyva A Salas-Espejo E Arffman RK. Association of Accelerometer-Determined Physical Activity and Sedentary Behavior with the gut microbiome in middle-aged women: a compositional data approach. Scand J Med Sci Sports. (2024) 34:e14689. doi: 10.1111/sms.14689, PMID: 38946228

[3] Lynch SV Pedersen O. The human intestinal microbiome in health and disease. N Engl J Med. (2016) 375:2369–79. doi: 10.1056/NEJMra1600266 27974040

[4] Baquero F Nombela C. The microbiome as a human organ. Clin Microbiol Infect. (2012) 18:2–4. doi: 10.1111/j.1469-0691.2012.03916.x 22647038

[5] Shreiner AB Kao JY Young VB. The gut microbiome in health and in disease. Curr Opin Gastroenterol. (2015) 31:69–75. doi: 10.1097/MOG.0000000000000139 25394236 PMC4290017

[6] Hou K Wu ZX Chen XY Wang JQ Zhang D Xiao C. Microbiota in health and diseases. Signal Transduct Target Ther. (2022) 7:135. doi: 10.1038/s41392-022-00974-4, PMID: 35461318 PMC9034083

[7] Milani C Duranti S Bottacini F Casey E Turroni F Mahony J. The first microbial colonizers of the human gut: composition, activities, and health implications of the infant gut microbiota. Microbiol Mol Biol Rev. (2017) 81:e00036–17. doi: 10.1128/MMBR.00036-17 PMC5706746 29118049

[8] Sebastián Domingo JJ Sánchez Sánchez C. From the intestinal flora to the microbiome. Rev Esp Enferm Dig. (2018) 110:51–6. doi: 10.17235/reed.2017.4947/2017, PMID: 29271225

[9] Gomes AC Hoffmann C Mota JF. The human gut microbiota: metabolism and perspective in obesity. Gut Microbes. (2018) 9:308–25. doi: 10.1080/19490976.2018.1465157, PMID: 29667480 PMC6219651

[10] Sila S Jelić M Trivić I Tambić Andrašević A Hojsak I Kolaček S. Altered gut microbiota is present in newly diagnosed pediatric patients with inflammatory bowel disease. J Pediatr Gastroenterol Nutr. (2020) 70:497–502. doi: 10.1097/MPG.0000000000002611, PMID: 31899727

[11] Barzak B Hankus K Parmar S Wozniak S. The effect of physical activity on gut microbiota. A review. Medical. J Cell Biol. (2022) 10:138–43. doi: 10.2478/acb-2022-0021

[12] Pedersini P Turroni S Villafañe JH. Gut microbiota and physical activity: is there an evidence-based link? Sci Total Environ. (2020) 727:138648. doi: 10.1016/j.scitotenv.2020.138648, PMID: 32498183

[13] Villafañe JH Drago L. What is the site of pain osteoarthritis? A triple gut-brain-joint microbioma axis. Clin Exp Rheumatol. (2019) 37 Suppl 122:20–1. PMID: 31820715

[14] Bermon S Petriz B Kajėnienė A Prestes J Castell L Franco OL. The microbiota: an exercise immunology perspective. Exerc Immunol Rev. (2015) 21:70–9. PMID: 25825908

[15] Durk RP Castillo E Márquez-Magaña L Grosicki GJ Bolter ND Lee CM. Gut microbiota composition is related to cardiorespiratory fitness in healthy Young adults. Int J Sport Nutr Exerc Metab. (2019) 29:249–53. doi: 10.1123/ijsnem.2018-0024, PMID: 29989465 PMC6487229

[16] Rivière A Selak M Lantin D Leroy F De Vuyst L. Bifidobacteria and butyrate-producing Colon Bacteria: importance and strategies for their stimulation in the human gut. Front Microbiol. (2016) 7:979. doi: 10.3389/fmicb.2016.00979 27446020 PMC4923077

[17] Wade WG. The oral microbiome in health and disease. Pharmacol Res. (2013) 69:137–43. doi: 10.1016/j.phrs.2012.11.006 23201354

[18] Moye ZD Zeng L Burne RA. Fueling the caries process: carbohydrate metabolism and gene regulation by *Streptococcus mutans*. J Oral Microbiol. (2014) 6:6. doi: 10.3402/jom.v6.24878 PMC4157138 25317251

[19] Slocum C Kramer C Genco CA. Immune dysregulation mediated by the oral microbiome: potential link to chronic inflammation and atherosclerosis. J Intern Med. (2016) 280:114–28. doi: 10.1111/joim.12476, PMID: 26791914

[20] Yang Y Cai Q Zheng W Steinwandel M Blot WJ Shu XO. Oral microbiome and obesity in a large study of low-income and African-American populations. J Oral Microbiol. (2019) 11:1650597. doi: 10.1080/20002297.2019.1650597, PMID: 31489128 PMC6713186

[21] Figuero E Lindahl C Marín MJ Renvert S Herrera D Ohlsson O. Quantification of periodontal pathogens in vascular, blood, and subgingival samples from patients with peripheral arterial disease or abdominal aortic aneurysms. J Periodontol. (2014) 85:1182–93. doi: 10.1902/jop.2014.130604 24502612

[22] Mervish NA Hu J Hagan LA Arora M Frau C Choi J. Associations of the Oral microbiota with obesity and menarche in Inner City girls. J Child Obes. (2019) 4:2. doi: 10.36648/2572-5394.4.1.68 31535093 PMC6750217

[23] Dalla Vecchia CF Susin C Rösing CK Oppermann RV Albandar JM. Overweight and obesity as risk indicators for periodontitis in adults. J Periodontol. (2005) 76:1721–8. doi: 10.1902/jop.2005.76.10.1721 16253094

[24] Braundmeier AG Lenz KM Inman KS Chia N Jeraldo P Walther-António MRS. Individualized medicine and the microbiome in reproductive tract. Front Physiol. (2015) 6:97. doi: 10.3389/fphys.2015.00097 25883569 PMC4381647

[25] Haffajee AD Socransky SS. Relation of body mass index, periodontitis and *Tannerella forsythia*. J Clin Periodontol. (2009) 36:89–99. doi: 10.1111/j.1600-051X.2008.01356.x, PMID: 19207883

[26] Fan X Alekseyenko AV Wu J Peters BA Jacobs EJ Gapstur SM. Human oral microbiome and prospective risk for pancreatic cancer: a population-based nested case-control study. Gut. (2018) 67:120–7. doi: 10.1136/gutjnl-2016-312580, PMID: 27742762 PMC5607064

[27] Hayes RB Ahn J Fan X Peters BA Ma Y Yang L. Association of Oral Microbiome with Risk for incident head and neck squamous cell Cancer. JAMA Oncol. (2018) 4:358–65. doi: 10.1001/jamaoncol.2017.4777, PMID: 29327043 PMC5885828

[28] Flemer B Warren RD Barrett MP Cisek K Das A Jeffery IB. The oral microbiota in colorectal cancer is distinctive and predictive. Gut. (2018) 67:1454–63. doi: 10.1136/gutjnl-2017-314814, PMID: 28988196 PMC6204958

[29] Yang Y Cai Q Shu XO Steinwandel MD Blot WJ Zheng W. Prospective study of oral microbiome and colorectal cancer risk in low-income and African American populations. Int J Cancer. (2019) 144:2381–9. doi: 10.1002/ijc.31941, PMID: 30365870 PMC6430704

[30] Zhang X Li L Butcher J Stintzi A Figeys D. Advancing functional and translational microbiome research using meta-omics approaches. Microbiome. (2019) 7:154. doi: 10.1186/s40168-019-0767-6, PMID: 31810497 PMC6898977

[31] Dorelli B Gallè F De Vito C Duranti G Iachini M Zaccarin M. Can physical activity influence human gut microbiota composition independently of diet? A systematic review. Nutrients. (2021) 13:1890. doi: 10.3390/nu13061890 34072834 PMC8228232

[32] Sabater C Iglesias-Gutiérrez E Ruiz L Margolles A. Next-generation sequencing of the athletic gut microbiota: a systematic review. Microbiome Res Rep. (2023) 2:5. doi: 10.20517/mrr.2022.16, PMID: 38045609 PMC10688803

[33] Donati Zeppa S Agostini D Gervasi M Annibalini G Amatori S Ferrini F. Mutual interactions among exercise, sport supplements and microbiota. Nutrients. (2019) 12:17. doi: 10.3390/nu12010017, PMID: 31861755 PMC7019274

[34] Monda V Villano I Messina A Valenzano A Esposito T Moscatelli F. Exercise modifies the gut microbiota with positive health effects. Oxidative Med Cell Longev. (2017) 2017:3831972. doi: 10.1155/2017/3831972 PMC5357536 28357027

[35] Clauss M Gérard P Mosca A Leclerc M. Interplay between exercise and gut microbiome in the context of human health and performance. Front Nutr. (2021) 8:637010. doi: 10.3389/fnut.2021.637010, PMID: 34179053 PMC8222532

[36] Barton W Penney NC Cronin O Garcia-Perez I Molloy MG Holmes E. The microbiome of professional athletes differs from that of more sedentary subjects in composition and particularly at the functional metabolic level. Gut. (2018) 67:625–33. doi: 10.1136/gutjnl-2016-313627, PMID: 28360096

[37] Quiroga R Nistal E Estébanez B Porras D Juárez-Fernández M Martínez-Flórez S. Exercise training modulates the gut microbiota profile and impairs inflammatory signaling pathways in obese children. Exp Mol Med. (2020) 52:1048–61. doi: 10.1038/s12276-020-0459-0, PMID: 32624568 PMC8080668

[38] Bai J Hu Y Bruner DW. Composition of gut microbiota and its association with body mass index and lifestyle factors in a cohort of 7-18 years old children from the American gut project. Pediatr Obes. (2019) 14:e12480. doi: 10.1111/ijpo.12480 30417607

[39] Moran-Ramos S Lopez-Contreras BE Villarruel-Vazquez R Ocampo-Medina E Macias-Kauffer L Martinez-Medina JN. Environmental and intrinsic factors shaping gut microbiota composition and diversity and its relation to metabolic health in children and early adolescents: a population-based study. Gut Microbes. (2020) 11:900–17. doi: 10.1080/19490976.2020.1712985, PMID: 31973685 PMC7524342

[40] Santarossa S Sitarik AR Johnson CC Li J Lynch SV Ownby DR. Associations of physical activity with gut microbiota in pre-adolescent children. Phys Act Nutr. (2021) 25:24–37. doi: 10.20463/pan.2021.0023, PMID: 35152621 PMC8843867

[41] Mancini A Cerulli C Vitucci D Lasorsa VA Parente D Di Credico A. Impact of active lifestyle on the primary school children saliva microbiota composition. Front Nutr. (2023) 10:1226891. doi: 10.3389/fnut.2023.1226891, PMID: 37671197 PMC10476528

[42] Lupo C De Pasquale P Boccia G Ungureanu AN Moisè P Mulasso A. The Most active child is not always the fittest: physical activity and fitness are weakly correlated. Sports (Basel). (2022) 11:3. doi: 10.3390/sports11010003, PMID: 36668707 PMC9866618

[43] Centro Epi. Le nuove curve di crescita dell’Oms: una riflessione. (2006) Available from: https://www.epicentro.iss.it/materno/curve_crescita (Accessed July 3, 2024).

[44] Twisk JWR. Physical activity guidelines for children and adolescents: a critical review. Sports Med. (2001) 31:617–27. doi: 10.2165/00007256-200131080-00006 11475323

[45] Hands B. How fundamental are fundamental movement skills? Active and Healthy Lifestyle Magazine. (2012) 1:14–7.

[46] Services D of H & H. Resistance training – health benefits. (2022). Department of Health & Human Services; Available from: http://www.betterhealth.vic.gov.au/health/healthyliving/resistance-training-health-benefits (Accessed July 3, 2024).

[47] lagudu sanjana. Mom Junction. (2015). Aerobics for kids: 10 benefits and 15 exercises. Available from: https://www.momjunction.com/articles/benefits-of-aerobic-exercises-for-kids_00335602/ (Accessed July 3, 2024).

[48] EUROFIT. Handbook for the eurofit tests of physical fitness. Rome: Committee for the Development of Sport, Council of Europe.

[49] Condon C Cremin K. Static balance norms in children. Physiother Res Int. (2014) 19:1–7. doi: 10.1002/pri.1549, PMID: 23703740

[50] Armstrong N Welsman J. Comment on ‘developing a new curvilinear Allometric model to improve the fit and validity of the 20-m shuttle run test as a predictor of cardiorespiratory fitness in adults and youth’. Sports Med. (2021) 51:1591–3. doi: 10.1007/s40279-021-01462-5, PMID: 34014502

[51] De Miguel-Etayo P Gracia-Marco L Ortega FB Intemann T Foraita R Lissner L. Physical fitness reference standards in European children: the IDEFICS study. Int J Obes. (2014) 38:S57–66. doi: 10.1038/ijo.2014.136 25376221

[52] Roth A Schmidt SCE Seidel I Woll A Bös K. Tracking of physical fitness of primary school children in Trier: a 4-year longitudinal study. Biomed Res Int. (2018) 2018:1–10. doi: 10.1155/2018/7231818 PMC5937626 29850555

[53] Granacher U Borde R. Effects of sport-specific training during the early stages of Long-term athlete development on physical fitness, body composition, cognitive, and academic performances. Front Physiol. (2017) 8:810. doi: 10.3389/fphys.2017.00810, PMID: 29085304 PMC5650693

[54] Callahan BJ McMurdie PJ Rosen MJ Han AW Johnson AJA Holmes SP. DADA2: high-resolution sample inference from Illumina amplicon data. Nat Methods. (2016) 13:581–3. doi: 10.1038/nmeth.3869, PMID: 27214047 PMC4927377

[55] Pruesse E Quast C Knittel K Fuchs BM Ludwig W Peplies J. SILVA: a comprehensive online resource for quality checked and aligned ribosomal RNA sequence data compatible with ARB. Nucleic Acids Res. (2007) 35:7188–96. doi: 10.1093/nar/gkm864, PMID: 17947321 PMC2175337

[56] McMurdie PJ Holmes S. Phyloseq: an R package for reproducible interactive analysis and graphics of microbiome census data. PLoS One. (2013) 8:e61217. doi: 10.1371/journal.pone.0061217, PMID: 23630581 PMC3632530

[57] Paradis E Schliep K. Ape 5.0: an environment for modern phylogenetics and evolutionary analyses in R. Bioinformatics. (2019) 35:526–8. doi: 10.1093/bioinformatics/bty633, PMID: 30016406

[58] Davis NM Proctor DM Holmes SP Relman DA Callahan BJ. Simple statistical identification and removal of contaminant sequences in marker-gene and metagenomics data. Microbiome. (2018) 6:226. doi: 10.1186/s40168-018-0605-2, PMID: 30558668 PMC6298009

[59] Dhariwal A Chong J Habib S King IL Agellon LB Xia J. Microbiome analyst: a web-based tool for comprehensive statistical, visual and meta-analysis of microbiome data. Nucleic Acids Res. (2017) 45:W180–8. doi: 10.1093/nar/gkx295, PMID: 28449106 PMC5570177

[60] Chong J Liu P Zhou G Xia J. Using microbiome analyst for comprehensive statistical, functional, and meta-analysis of microbiome data. Nat Protoc. (2020) 15:799–821. doi: 10.1038/s41596-019-0264-1, PMID: 31942082

[61] Mancini A Martone D Vitucci D Capobianco A Alfieri A Buono P. Influence of sport practice and body weight on physical fitness in schoolchildren living in the Campania region. Int J Environ Res Public Health. (2022) 19:7412. doi: 10.3390/ijerph19127412, PMID: 35742659 PMC9223406

[62] Segata N Izard J Waldron L Gevers D Miropolsky L Garrett WS. Metagenomic biomarker discovery and explanation. Genome Biol. (2011) 12:R60. doi: 10.1186/gb-2011-12-6-r60, PMID: 21702898 PMC3218848

[63] Shaw L Ribeiro ALR Levine AP Pontikos N Balloux F Segal AW. The human salivary microbiome is shaped by shared environment rather than genetics: evidence from a large family of closely related individuals. MBio. (2017) 8:e01237–17. doi: 10.1128/mBio.01237-17 28900019 PMC5596345

[64] Sovran B Hugenholtz F Elderman M Van Beek AA Graversen K Huijskes M. Age-associated impairment of the mucus barrier function is associated with profound changes in microbiota and immunity. Sci Rep. (2019) 9:1437. doi: 10.1038/s41598-018-35228-3, PMID: 30723224 PMC6363726

[65] Ballini A Scacco S Boccellino M Santacroce L Arrigoni R. Microbiota and obesity: where are we now? Biology (Basel). (2020) 9:415. doi: 10.3390/biology9120415 33255588 PMC7761345

[66] Asadi A Shadab Mehr N Mohamadi MH Shokri F Heidary M Sadeghifard N. Obesity and gut-microbiota-brain axis: a narrative review. J Clin Lab Anal. (2022) 36:e24420. doi: 10.1002/jcla.24420, PMID: 35421277 PMC9102524

[67] Lay C Sutren M Rochet V Saunier K Doré J Rigottier-Gois L. Design and validation of 16S rRNA probes to enumerate members of the *Clostridium leptum* subgroup in human faecal microbiota. Environ Microbiol. (2005) 7:933–46. doi: 10.1111/j.1462-2920.2005.00763.x 15946290

[68] Cataldi S Poli L Şahin FN Patti A Santacroce L Bianco A. The effects of physical activity on the gut microbiota and the gut–brain Axis in preclinical and human models: a narrative review. Nutrients. (2022) 14:3293. doi: 10.3390/nu14163293, PMID: 36014798 PMC9413457

[69] Motiani KK Collado MC Eskelinen JJ Virtanen KA Löyttyniemi E Salminen S. Exercise training modulates gut microbiota profile and improves Endotoxemia. Med Sci Sports Exerc. (2020) 52:94–104. doi: 10.1249/MSS.0000000000002112, PMID: 31425383 PMC7028471

[70] Cataldi S Bonavolontà V Poli L Clemente FM De Candia M Carvutto R. The relationship between physical activity, physical exercise, and human gut microbiota in healthy and unhealthy subjects: a systematic review. Biology (Basel). (2022) 11:479. doi: 10.3390/biology11030479 35336852 PMC8945171

[71] Aragón-Vela J Solis-Urra P Ruiz-Ojeda FJ Álvarez-Mercado AI Olivares-Arancibia J Plaza-Diaz J. Impact of exercise on gut microbiota in obesity. Nutrients. (2021) 13:3999. doi: 10.3390/nu13113999, PMID: 34836254 PMC8624603

[72] Ramos C Gibson GR Walton GE Magistro D Kinnear W Hunter K. Systematic review of the effects of exercise and physical activity on the gut microbiome of older adults. Nutrients. (2022) 14:674. doi: 10.3390/nu14030674, PMID: 35277033 PMC8837975

[73] Louis P Flint HJ. Diversity, metabolism and microbial ecology of butyrate-producing bacteria from the human large intestine. FEMS Microbiol Lett. (2009) 294:1–8. doi: 10.1111/j.1574-6968.2009.01514.x 19222573

[74] De Filippis F Pasolli E Ercolini D. Newly explored Faecalibacterium diversity is connected to age, lifestyle, geography, and disease. Curr Biol. (2020) 30:4932–4943.e4. doi: 10.1016/j.cub.2020.09.063 33065016

[75] Roberts JD Suckling CA Peedle GY Murphy JA Dawkins TG Roberts MG. An exploratory investigation of endotoxin levels in novice Long distance triathletes, and the effects of a multi-strain probiotic/prebiotic, antioxidant intervention. Nutrients. (2016) 8:733. doi: 10.3390/nu8110733, PMID: 27869661 PMC5133117

[76] Wang J Zhang Q Xia J Sun H. Moderate treadmill exercise modulates gut microbiota and improves intestinal barrier in high-fat-diet-induced obese mice via the AMPK/CDX2 signaling pathway. Diabetes Metab Syndr Obes. (2022) 15:209–23. doi: 10.2147/DMSO.S346007, PMID: 35087282 PMC8789310

[77] Estaki M Pither J Baumeister P Little JP Gill SK Ghosh S. Cardiorespiratory fitness as a predictor of intestinal microbial diversity and distinct metagenomic functions. Microbiome. (2016) 4:42. doi: 10.1186/s40168-016-0189-7, PMID: 27502158 PMC4976518

[78] McNamara MP Singleton JM Cadney MD Ruegger PM Borneman J Garland T. Early-life effects of juvenile Western diet and exercise on adult gut microbiome composition in mice. J Exp Biol. (2021) 224:jeb 239699. doi: 10.1242/jeb.239699, PMID: 33431595 PMC7929929

[79] Ridaura VK Faith JJ Rey FE Cheng J Duncan AE Kau AL. Gut microbiota from twins discordant for obesity modulate metabolism in mice. Science. (2013) 341:1241214. doi: 10.1126/science.1241214, PMID: 24009397 PMC3829625

[80] Mika A Van Treuren W González A Herrera JJ Knight R Fleshner M. Exercise is more effective at altering gut microbial composition and producing stable changes in lean mass in juvenile versus adult male F344 rats. PLoS One. (2015) 10:e0125889. doi: 10.1371/journal.pone.0125889, PMID: 26016739 PMC4446322

[81] Yatsunenko T Rey FE Manary MJ Trehan I Dominguez-Bello MG Contreras M. Human gut microbiome viewed across age and geography. Nature. (2012) 486:222–7. doi: 10.1038/nature11053, PMID: 22699611 PMC3376388

[82] Ortega FB Artero EG Ruiz JR España-Romero V Jiménez-Pavón D Vicente-Rodriguez G. Physical fitness levels among European adolescents: the HELENA study. Br J Sports Med. (2011) 45:20–9. doi: 10.1136/bjsm.2009.062679 19700434

[83] Ortega FB Leskošek B Blagus R Gil-Cosano JJ Mäestu J Tomkinson GR. European fitness landscape for children and adolescents: updated reference values, fitness maps and country rankings based on nearly 8 million test results from 34 countries gathered by the fit Back network. Br J Sports Med. (2023) 57:299–310. doi: 10.1136/bjsports-2022-106176, PMID: 36623866 PMC9985767

[84] Raju SC Lagström S Ellonen P de Vos WM Eriksson JG Weiderpass E. Gender-specific associations between saliva microbiota and body size. Front Microbiol. (2019) 10. doi: 10.3389/fmicb.2019.00767 PMC6467948 31024514

[85] Segata N Haake SK Mannon P Lemon KP Waldron L Gevers D. Composition of the adult digestive tract bacterial microbiome based on seven mouth surfaces, tonsils, throat and stool samples. Genome Biol. (2012) 13:R42. doi: 10.1186/gb-2012-13-6-r42, PMID: 22698087 PMC3446314

[86] Tett A Pasolli E Masetti G Ercolini D Segata N. Prevotella diversity, niches and interactions with the human host. Nat Rev Microbiol. (2021) 19:585–99. doi: 10.1038/s41579-021-00559-y, PMID: 34050328 PMC11290707

[87] Ortega-Santos CP Whisner CM. The key to successful weight loss on a high-Fiber diet may be in gut microbiome Prevotella abundance. J Nutr. (2019) 149:2083–4. doi: 10.1093/jn/nxz248, PMID: 31584088

[88] Hjorth MF Roager HM Larsen TM Poulsen SK Licht TR Bahl MI. Pre-treatment microbial Prevotella-to-Bacteroides ratio, determines body fat loss success during a 6-month randomized controlled diet intervention. Int J Obes. (2018) 42:284. doi: 10.1038/ijo.2018.1, PMID: 29406520 PMC7609312

[89] Larsen JM. The immune response to Prevotella bacteria in chronic inflammatory disease. Immunology. (2017) 151:363–74. doi: 10.1111/imm.12760, PMID: 28542929 PMC5506432

[90] Christensen L Roager HM Astrup A Hjorth MF. Microbial enterotypes in personalized nutrition and obesity management. Am J Clin Nutr. (2018) 108:645–51. doi: 10.1093/ajcn/nqy175, PMID: 30239555

[91] Hjorth MF Blædel T Bendtsen LQ Lorenzen JK Holm JB Kiilerich P. Prevotella-to-Bacteroides ratio predicts body weight and fat loss success on 24-week diets varying in macronutrient composition and dietary fiber: results from a post-hoc analysis. Int J Obes. (2019) 43:149–57. doi: 10.1038/s41366-018-0093-2, PMID: 29777234 PMC6331389

[92] Chung WSF Walker AW Bosscher D Garcia-Campayo V Wagner J Parkhill J. Relative abundance of the Prevotella genus within the human gut microbiota of elderly volunteers determines the inter-individual responses to dietary supplementation with wheat bran arabinoxylan-oligosaccharides. BMC Microbiol. (2020) 20:283. doi: 10.1186/s12866-020-01968-4, PMID: 32928123 PMC7490872

[93] Kovatcheva-Datchary P Nilsson A Akrami R Lee YS De Vadder F Arora T. Dietary Fiber-induced improvement in glucose metabolism is associated with increased abundance of Prevotella. Cell Metab. (2015) 22:971–82. doi: 10.1016/j.cmet.2015.10.001, PMID: 26552345

[94] Garber JM Hennet T Szymanski CM. Significance of fucose in intestinal health and disease. Mol Microbiol. (2021) 115:1086–93. doi: 10.1111/mmi.14681 33434389

[95] Steinchen W Zegarra V Bange G. (p)pp Gpp: magic modulators of bacterial physiology and metabolism. Front Microbiol. (2020) 11:2072. doi: 10.3389/fmicb.2020.02072, PMID: 33013756 PMC7504894

[96] Dai L Mafra D Shiels PG Hackeng TM Stenvinkel P Schurgers LJ. Vitamin K and hallmarks of ageing: focus on diet and gut microbiome. Nutrients. (2023) 15:2727. doi: 10.3390/nu15122727, PMID: 37375631 PMC10301624

[97] Shea MK Barger K Booth SL Matuszek G Cushman M Benjamin EJ. Vitamin K status, cardiovascular disease, and all-cause mortality: a participant-level meta-analysis of 3 US cohorts. Am J Clin Nutr. (2020) 111:1170–7. doi: 10.1093/ajcn/nqaa082, PMID: 32359159 PMC7266692

[98] Geleijnse JM Vermeer C Grobbee DE Schurgers LJ Knapen MHJ van der Meer IM. Dietary intake of menaquinone is associated with a reduced risk of coronary heart disease: the Rotterdam study. J Nutr. (2004) 134:3100–5. doi: 10.1093/jn/134.11.3100, PMID: 15514282

[99] Orrù S Imperlini E Vitucci D Caterino M Mandola A Randers MB. Insight into the molecular signature of skeletal muscle characterizing lifelong football players. Int J Environ Res Public Health. (2022) 19:15835. doi: 10.3390/ijerph192315835, PMID: 36497910 PMC9740844

[100] Stahringer SS Clemente JC Corley RP Hewitt J Knights D Walters WA. Nurture trumps nature in a longitudinal survey of salivary bacterial communities in twins from early adolescence to early adulthood. Genome Res. (2012) 22:2146–52. doi: 10.1101/gr.140608.112, PMID: 23064750 PMC3483544

[101] Yi W Xinxin P Xiaoya L Dandan L Zhoujin T Rong Y. Sex hormones influence the intestinal microbiota composition in mice. Front Microbiol. (2022) 13:964847. doi: 10.3389/fmicb.2022.964847 36386696 PMC9659915

