# Supplementary figures and images for: Six months of different exercise type in sedentary primary schoolchildren: impact on physical fitness and saliva microbiota composition
Source: Front Nutr. 2024 Oct 24;11:1465707. doi: 10.3389/fnut.2024.1465707 (PMC11542257; doi:10.3389/fnut.2024.1465707)

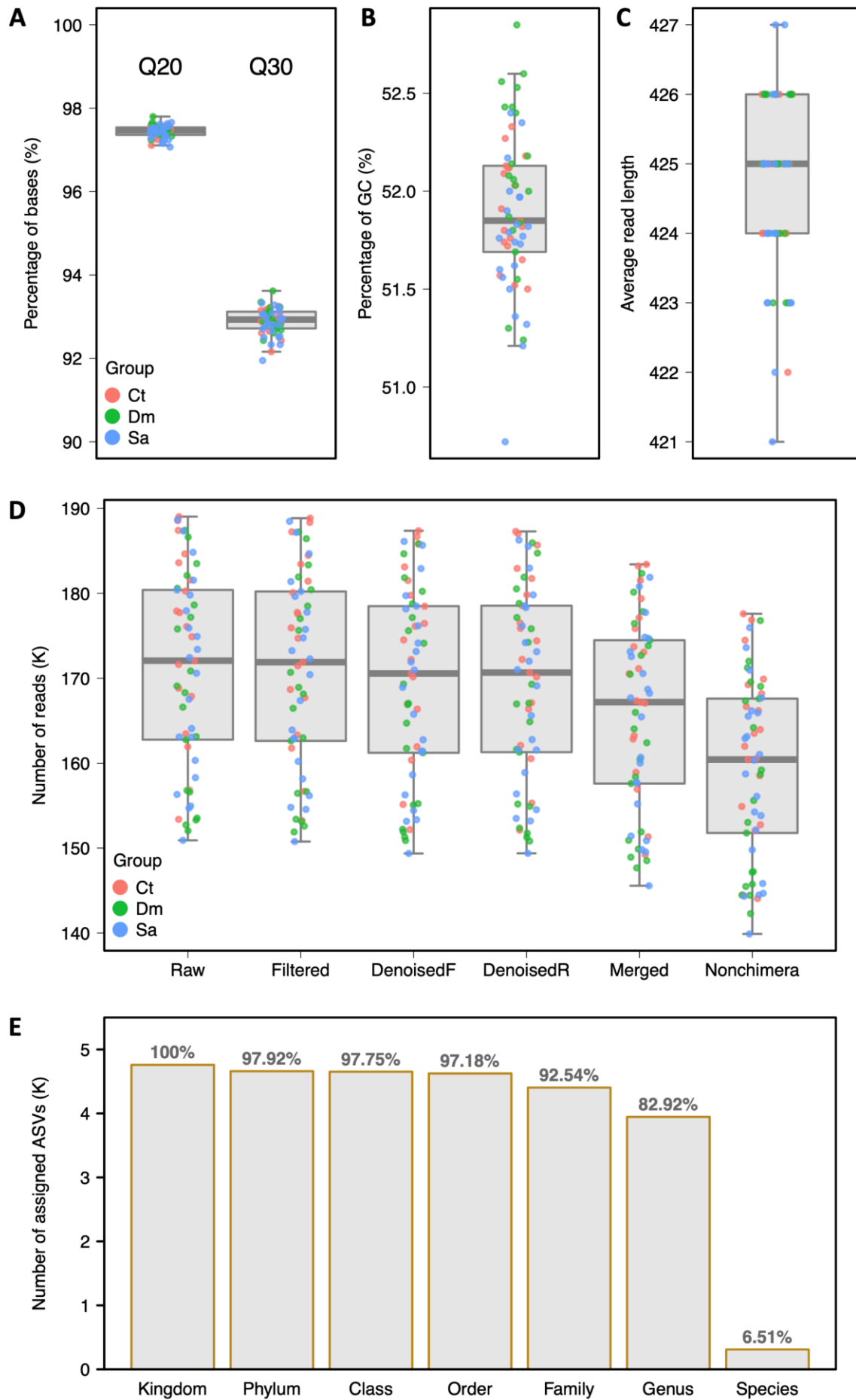

Supplement: Supplementary file 1 [file Image_1.pdf]
